# Establishing hematopoietic stem cell transplant programs; overcoming cost through collaboration

**DOI:** 10.1038/s41409-020-0793-9

**Published:** 2020-01-21

**Authors:** Hani Al-Hashmi, Ahmed Alsagheir, Analie Estanislao, Jenifer Bacal, Ashraf Alsuhebah, Belal Alblowe, Heba Raslan, Arwa Alsaber, Ahmed Albahrani, Norah Almulhem, Amal Alamri, Khalid Alsaleh, Amal Albeihany, Amal Alabdulwahab, Mohammed Bakkar, Panayotis Kaloyannidis

**Affiliations:** 1 Oncology Center, Adult Hematology & Stem Cell Transplantation Department, King Fahad Specialist Hospital, P.O. Box 15373, Dammam, 31444 Saudi Arabia; 2 Oncology Center, John Hopkins Aramco Healthcare, Dhahran, 31311 Saudi Arabia; 3 Faculty of Medicine, King Saud University, P.O Box 2925, Riyadh, 11461 Saudi Arabia; 4 Department of Medicine, King Fahad General Hospital, Madinah, 42351 Saudi Arabia; 5 Oncology Center, King Abdullah Medical City, P.O. Box 57657, Makkah, 21955 Saudi Arabia; 6 Oncology Department, Prince Mohamed Bin Nasser Hospital, Jizan, 82943 Saudi Arabia

**Keywords:** Health services, Haematopoietic stem cells

Hematopoietic stem cell transplantation (HSCT) has dramatically evolved since the 1960s [1, 2]. It requires trained and experienced medical staffs and fully equipped centers to offer optimal care. HSCT cost data in different part of the world is ranging from 12,500 US $ in Mexico to 549,208 US $ total health care cost of myeloablative allogeneic HSCT at 1 year in the United States. However, published details on the establishment cost of HSCT programs are limited [3, 4]. Centralizing such specialized service in few centers could negatively affect the operating cost and might result in staff burnout, long waiting lists that could lead to poor outcomes [5]. In an era of global financial constraints, innovative approaches to reach cost efficiency and optimal clinical outcomes still not readily available [6]. We sought to establish a collaborative process to expand the HSCT services in Saudi Arabia (KSA).

We used the available data on the population densities and services availabilities across KSA to guide our initiative. We have decided to start with autologous HSCT program to build team knowledge, familiarity, and confidence with the process of HSCT as well as the economic feasibility compared to allogeneic HSCT [7]. At the beginning, experienced team of physicians, pharmacists, nurses and technicians (core team) met to discuss the need for such initiative and planed a stepwise approach to assist new centers. Four phases were planed; assessment, planning/training, site activation, and the final phase were to ensure the continuous support and improvement. We visited the clinical, pharmacy, and laboratory areas in selected centers to assess their current capabilities, which were fundamental in identifying strength, opportunities as well as the required support and training [8]. Assessment confirmed that all hospitals with chemotherapy capabilities could fulfill the minimal required criteria for establishing HSCT program as per the Worldwide Network for Blood and Marrow Transplantation recommendation [9]. We utilized this information to organize the required theoretical and hands-on training as well as logistics support. The champion in-charge of each new center identified his/her potential team members. The most important phase is planning/training and should not be rushed, since it is considered the foundation for all future activities. Quality and administrative training were done to ensure familiarity with forms, documentation and to avoid non-conformances that could result in suboptimal quality and cost inefficiency. The new centers screened potential patients and once identified, pre-transplant process, forms and consents were completed and road-maps were generated with the support of the core team and approved by the hematologist at the new center to signal site activation. This phase that would be completed by infusing the stem cells was followed by supportive care period that was characterized by full communication between the core team and the new center’s team and facilitated through a WhatsApp group. The core team’s involvement in the subsequent transplants was as per the new center discretion. All centers were encouraged to participate in an annual HSCT activity to share experiences and best practices.

**Fig. 1:**
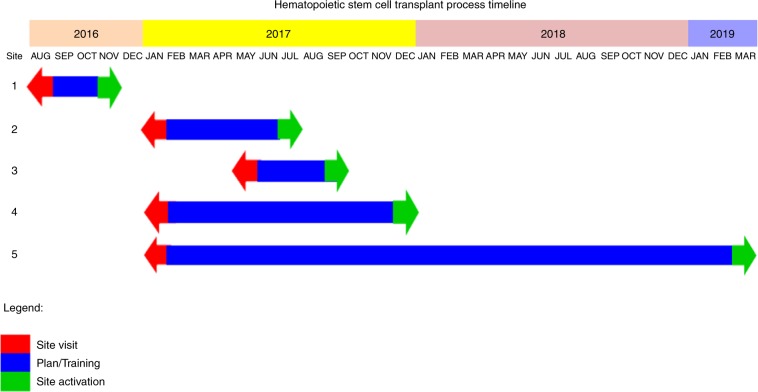
Project time-line.

Change management and stakeholder’s alignment are essential for successful establishment of such program. This was very clear in center 5 were a lot of effort and time were taken to engage stakeholders and manage internal resistance (Fig. 1). Initially, we faced resistance that slowly resolved by creating a culture for the change required through aligning the patients’ needs and the team ambitious goal. We focused on improving the communication platform, and celebrating success at every step. We also held a number of lessons learned sessions with the core team and the new centers to optimize the establishment process. Although, traditional communication style through regular meeting and emails are essential to discuss the details of the plan and the process, however, new technologies and applications will enhance medical communication in this era. In our project, this was fairly evident during the site activation phase since we used WhatsApp application as an additional tool to improve communication and promote faster and real-time discussion. We believe that smart solutions and technologies will greatly impact health care delivery and outcomes. This approach helped in establishing 5 new HSCT centers with average cost saving exceeded 4 million US $ per center. We also introduced to our health care system an independent teams and health care providers who could contribute to future collaborations. We believe such work could be replicated to support optimal health care delivery and cost efficiency in any health care system.

Health care organizations embarking on such projects will be faced with financial constraints and limited with minimal start-up experiences. Empowering new programs though collaboration have many advantages such as overcoming multiple financial limitations, shortening the project duration and simplifying any processes benefiting from the cumulative experiences to avoid waste and non-value adding steps as well as identifying all the establishment cost saving opportunities [10]. It also gives a broader platform to apply business improvement methodologies that have been tested in different industries (manufacturing, telecommunication, aviation, etc) such as Lean Six Sigma (LSS) in the health care industry.
